# An immunoproteomic approach revealing peptides from *Sporothrix brasiliensis* that induce a cellular immune response in subcutaneous sporotrichosis

**DOI:** 10.1038/s41598-018-22709-8

**Published:** 2018-03-08

**Authors:** José Roberto Fogaça de Almeida, Grasielle Pereira Jannuzzi, Gilberto Hideo Kaihami, Leandro Carvalho Dantas Breda, Karen Spadari Ferreira, Sandro Rogério de Almeida

**Affiliations:** 1 Faculty of Pharmaceutical Sciences, Department of Clinical and Toxicological Analysis, University of Sao Paulo, Sao Paulo, Brazil; 2 Institute of Chemistry, Department of Biochemistry, University of Sao Paulo, Sao Paulo, Brazil; 3 Department of Immunology, Institute of Biomedical Sciences, University of São Paulo, São Paulo, Brazil; 4 Department of Biological Sciences, Institute of Environmental, Chemical and Pharmaceutical Sciences, Federal University of São Paulo, Diadema, Brazil

## Abstract

*Sporothrix brasiliensis* is the most virulent fungus of the *Sporothrix* complex and is the main species recovered in the sporotrichosis zoonotic hyperendemic area in Rio de Janeiro. A vaccine against *S*. *brasiliensis* could improve the current sporotrichosis situation. Here, we show 3 peptides from *S*. *brasiliensis* immunogenic proteins that have a higher likelihood for engaging MHC-class II molecules. We investigated the efficiency of the peptides as vaccines for preventing subcutaneous sporotrichosis. In this study, we observed a decrease in lesion diameters in peptide-immunized mice, showing that the peptides could induce a protective immune response against subcutaneous sporotrichosis. ZR8 peptide is from the GP70 protein, the main antigen of the *Sporothrix* complex, and was the best potential vaccine candidate by increasing CD4^+^ T cells and higher levels of IFN-γ, IL-17A and IL-1β characterizing a strong cellular immune response. This immune environment induced a higher number of neutrophils in lesions that are associated with fungus clearance. These results indicated that the ZR8 peptide induces a protective immune response against subcutaneous sporotrichosis and is a vaccine candidate against *S*. *brasiliensis* infection.

## Introduction

Sporotrichosis is a subcutaneous mycosis caused by dimorphic fungi from the *Sporothrix* complex^1–3^. The fungus enters the subcutaneous tissue through surface injuries from plant debris or cat scratches. The fungus commonly reaches the lymphatic system and causes a chronic development of erythematous nodules in subcutaneous tissue^4^. *Sporothrix* complex species are cosmopolitan fungi, and sporotrichosis is a worldwide disease frequently reported in Latin American countries^5^. The incidence of sporotrichosis in Brazil has increased^6^. Including human and animal cases, more than 8,000 cases of sporotrichosis were diagnosed in Rio de Janeiro between 1998 and 2012^7^. With fewer numbers of cases than Rio de Janeiro, similar sporotrichosis cases occurred in other Brazilian states as Rio Grande do Sul and São Paulo^8–10^. This endemic in Rio de Janeiro is associated with zoonotic transmission by infected cats, mostly by deep scratching or biting that inoculates high loads of *Sporothrix* spp. into the host tissue. *S*. *brasiliensis* is the most diagnosed species in this zoonotic endemic^8^. Among the species of the *Sporothrix* complex, *S*. *schenckii* and *S*. *globosa* are the current species associated with sapronotic sporotrichosis, and *S*. *brasiliensis* is associated with zoonotic sporotrichosis^3,6,11^. *S*. *brasiliensis* is the most virulent species of the complex in sporotrichosis models^12–16^. The severity of sporotrichosis varies with the host immune system and the *Sporothrix* species virulence. Both a healthy host and an immunosuppressed one can develop the lymphocutaneous form of sporotrichosis. However, immunosuppression predisposes the host to a higher tendency to develop disseminated and severe forms of sporotrichosis^17^.

In feline sporotrichosis, the treatment can use different drug protocols that include potassium iodide, itraconazole and amphotericin B^7^. The treatment may take several months and is very difficult due to the difficulty of giving medicine to cats that are stressed and can scratch their owners^7^. In mice, a sporotrichosis model treatment with mAb P6E7 (a monoclonal antibody against an antigenic 70 kDa fungal protein (GP70)) showed prophylactic and therapeutic activity against sporotrichosis caused by *S*. *schenckii* and *S*. *brasiliensis*^16,18^. A humanized mAb P6E7 was recently developed that was able to increase phagocytosis in human monocytes and reduce the fungal burden in a sporotrichosis model^19^. However, the increased sporotrichosis cases in cats and humans create a new paradigm of sporotrichosis. A new therapy is necessary to reduce the number of sporotrichosis cases.

A vaccine against sporotrichosis, mostly in cats, could improve this paradigm. However, the development of effective vaccines against fungi is very difficult. The genetic complexity, limited knowledge of the mechanisms of the anti-fungal drugs and a lack of a defined antigen are some of the reasons for not having an effective antifungal vaccine^20^. For an effective vaccine, a protective immune response is essential. This protective immune response requires the interaction between an antigen-presenting cell (APC) and T cells, T cell clonal expansion, and differentiation into effector cells^21^. Using an immunoproteomic approach, we identified antigenic proteins from *S*. *brasiliensis* and classified the peptides that can couple to MHC class II to develop an effective immune response.

Finally, we show that the antigenic peptides ZR3, ZR4 and ZR8 can induce proliferation in T cells sensitized by *S*. *brasiliensis*. We also demonstrated that treatment with these peptides decreased the diameter of lesions in subcutaneous sporotrichosis. ZR8 peptide is able to promote higher levels of cytokines (IFN-γ, IL-17A and IL-1β, in the lesions and increase CD4^+^ T cells in the lymph nodes and spleen. Together these data demonstrated the efficacy of these peptides as a vaccine that promotes a protective immune response against sporotrichosis that can help in sporotrichosis endemic in Brazil. It can also help in the development of novel therapeutic approaches against fungal infections.

## Results

### *S*. *brasiliensis* proteome and antigenic proteins

**Figure 1:**
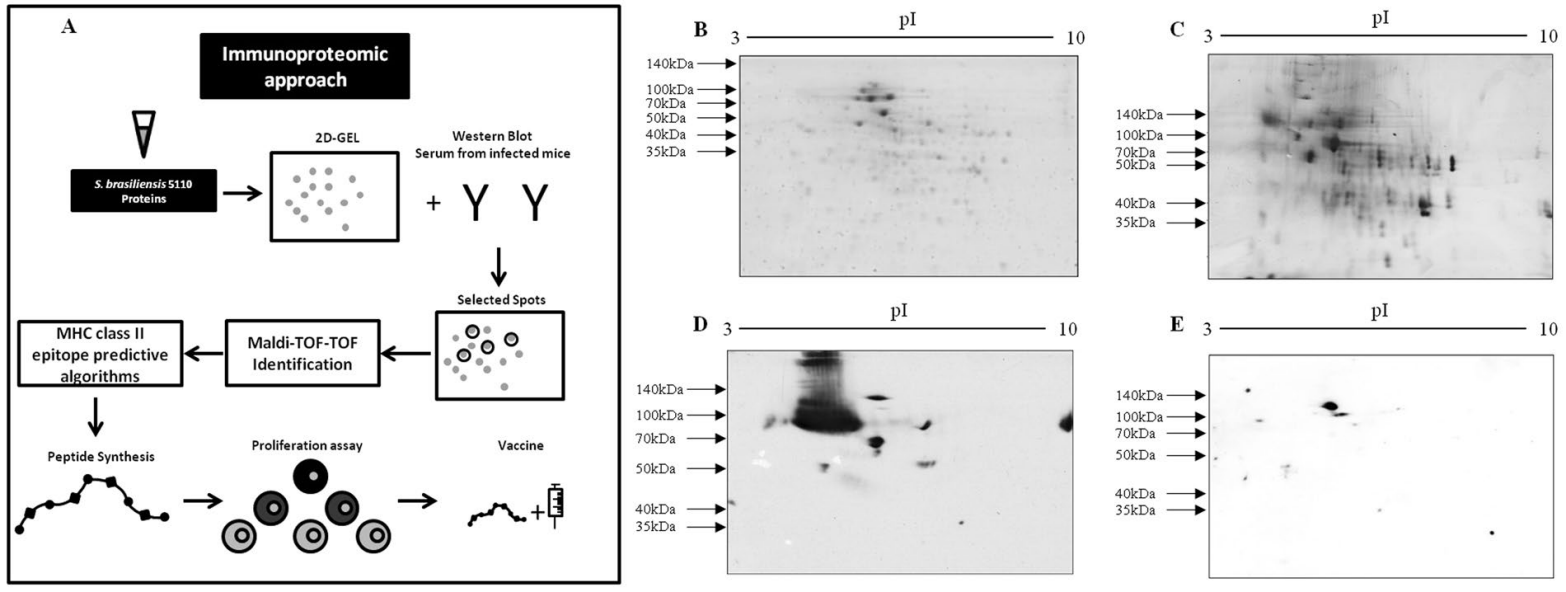
*S. brasiliensis* proteome and antigenic proteins. (**A**) Immunoproteomic approach elaborated. The fungus proteins were fractionated using 7 cm pH 3–10 (left to right) strips in the first dimension and 12% SDS-PAGE gels in the second dimension developed by (**B**) coomassie staining or (**C**) silver staining. (**D**) The spots recognized by western blot with sera from mice infected by *S*. *brasiliensis*. (**E**) The western blot with mAbP6E7.

To better analyze and identify the antigenic *Sporothrix brasiliensis* proteins, we used an immunoproteomic approach. A protocol was developed to use whole yeast cells to extract the fungal proteins (Fig. 1A). To evaluate the complexity of the *S*. *brasiliensis* 5110 proteins, 250 μg of proteins were fractionated by 2D electrophoresis. The 2D gel was stained by Coomassie blue and silver stains, and we observed the presence of 95 and 130 proteins, respectively (Fig. 1B and C). The proteins ranged from approximately 100 to 40 kDa with a pI of 4–5. These results are similar to those observed in other species of *Sporothrix* with different protein extraction protocols^22–24^. Two-dimensional Western blot analysis revealed 53 antigenic spots in the *S*. *brasiliensis* 5110 proteome with infected mouse serum. Most had molecular weights between 140 and 70 kDa with a pI of 4–5 (Fig. 1D). It was possible to observe isoforms of approximately 100 kDa. The presence of isoforms has already been seen in *S*. *brasiliensis* and other species from the *Sporothrix* complex^22,23^. The control serum showed few spots with low reactivity in the proteome of *S*. *brasiliensis* 5110 (data not shown). In contrast to infected mouse serum, human serum from patients with sporotrichosis revealed 11 spots in the proteome of environmental strain of *S*. *brasiliensis* (strain CBS 132990) that were in the range of 91 to 36 kDa^22^. The Western blot analysis performed with the mAb P6E7 identified a GP70 in the proteome of *S*. *brasiliensis* 5110 of approximately 100 kDa (Fig. 1E). A GP70 of similar weight has already been shown in *S*. *brasiliensis*^16^. The molecular weight of GP70 may vary due to glycosylation sites present in the GP70 of *S*. *brasiliensis* species^14^.

### Identification of the immunogenic proteins and peptides

**Figure 2:**
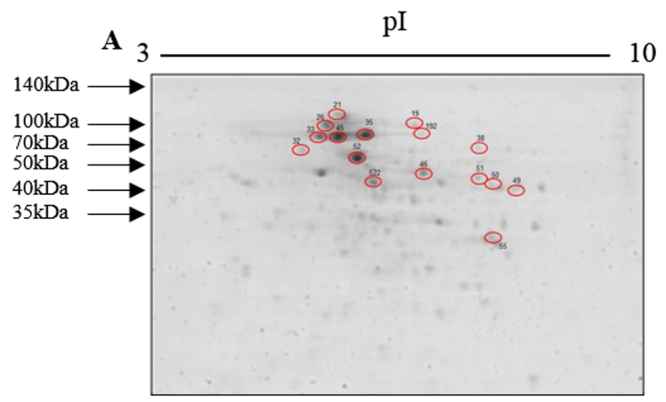
Selected spots. Selected spots to identification by MALDI–ToF MS/MS.

From 53 immunoreactive spots found in the Western blot, 16 spots were selected to be withdrawn from the Coomassie blue-stained 2D gel (Fig. 2A). The mass spectrometry data were paired with the approximately 9000 proteins of the *S*. *brasiliensis* 5110 genome^25^. We identified 34 immunogenic proteins from 14 spots, but 2 spots could not be identified (Table 1). GP70 was present in the *S*. *brasiliensis* proteome by mAbP6E7 Western blot but was not identified in the mass spectrometry. The identified proteins were related to many functions such as virulence, metabolic activities or unknown functions (Table 1). From the 34 proteins identified, 60 peptide sequences were considered with intermediate/high chances of engagement with the MHC class II molecules according to the parameters described in materials and methods. The 6 best scores in the prediction data were selected for synthesis (Table 2). A GP70 peptide, ZR8, was also synthesized. Although the prediction demonstrated that all GP70 peptide sequences have a low chance of engaging with MHC class II, GP70 is an important antigenic component of the *Sporothrix* complex^16,26,27^. From GP70, the sequence *LKFLALASVISATSA* was selected for synthesis for being the one with the greater chances of engagement with the MHC class II molecules.

**Table 1:** Immunogenic proteins identified in the *S*. *brasiliensis* 5110. The 34 immunogenic proteins identified in the proteome of *S*. *brasiliensis* 5110 (ATCC MYA 4823).

Spots	Protein	Coverage
ZR19	Aconitate hydratase RalA-binding protein 1	1.90% 0.62%
ZR21	Heat shock 70 kDa protein 4	1.11%
ZR26	Major facilitator superfamily transporter Molecular chaperone HtpG	1.33% 5.51%
ZR32	Hypothetic protein 1 Hypothetic protein 2	0.86% 0.85%
ZR33	Nuclear migration protein	0.68%
ZR35	C6 zinc finger domain containing protein Molecular chaperone DnaK Importin Hypothetic protein	1.13% 4.30% 1.18% 2.22%
ZR38	Hypothetic protein RalA-binding protein 1	0.86% 0.62%
ZR45	Heat shock 70 kDa protein 1/8	5.92%
ZR46	Hypothetic protein 1 Hypothetic protein 2 Aldehyde dehydrogenase (NAD^+^)	3.88% 4.49% 4.25%
ZR49	Cop9 subunit Hypothetic protein	1.46% 0.95%
ZR50	Not identified	—
ZR51	Histidyl-tRNA synthetase Hypothetic protein Mannose-1-phosphate Guanylyltransferase Autophagy protein	1.13% 2.85% 2.69% 0.61%
ZR52	Not identified	—
ZR55	Peptidyl-prolyl cis-trans isomerase-like 2 Hypothetic protein ABC transport system ATP-binding protein	1.32% 1.00% 0.91%
ZR192	Hypothetic protein MFS transporter Glutamate synthase (NADPH/NADH) Hypothetic protein1 Hypothetic protein 2	6.22% 1.77% 0.37% 4.41% 0.95%
**ZR522**	Enolase Hypothetic protein	3.42% 2.04%

**Table 2:** Selected peptides from S. *brasiliensis* 5110 proteome. The 7 selected peptides from the identified proteins in *S. brasiliensis* proteome. The peptides were selected by affinity to MHC class II using the Immune Epitope Database (IEDB) Analysis Resource and the PREDBALB/c. SMM align method (stabilization matrix alignment method); NN align method (neural network-based method).

Spot	Protein	Smm	nn	MHC	PREDBALB/c	Peptide	Peptide name
ZR55	Hypothetic protein	85	28	IAd	9.65	TRLMEMLAAQAALSN	**ZR1**
ZR35	Importin	158	88	IAd	9.60	QSHIMAMILSVQAAFP	**ZR3**
ZR51	Hypothetic protein	292	54	IAd	9.66	MSRTSSALKAVAAAET	**ZR4**
ZR32	Hypothetic protein 1	199	499	IAd	9.70	QMIVRIRAQLAEASR	**ZR5**
ZR55	Hypothetic protein	233	49	IAd	9.57	NEMRRRMMMESARDLE	**ZR6**
ZR32	Hypothetic protein 2	336	13	IAd	9.60	SVASYVAMQAVMDASR	**ZR7**
—	GP70	1308	331	IAd	8.98	LKFLALASVISATSA	**ZR8**

### The peptides ZR3, ZR4 and ZR8 were able to promote proliferation in *S*. *brasiliensis*-sensitized cells *in vitro*

**Figure 3:**
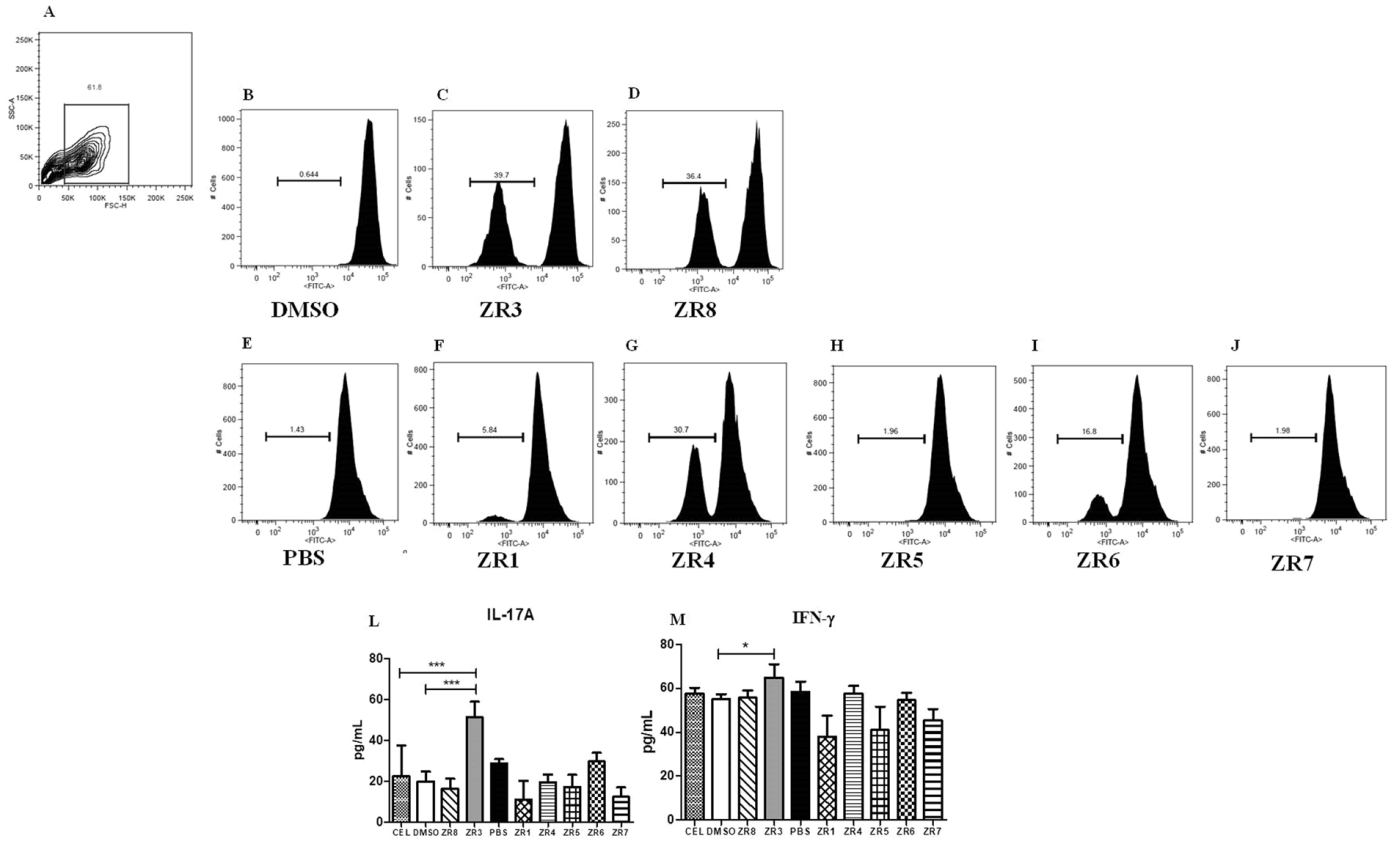
Cell expansion by peptides ZR3, ZR4 and ZR8 in *S*. *brasiliensis* sensitized cells *in vitro*. (**A**) The acquired population to exclude cellular debris. *In vitro* expansion of spleen cells labeled with CFSE with peptides and negative controls. (**B–D**) DMSO group and (**E–J**) PBS group. The cytokines levels of (**L**) IL-17A and (**M**) IFN-β in the supernatant were measured. Statistical analysis was performed using One-way ANOVA followed by Tukey’s test.

We evaluated whether the synthesized peptides could promote cell expansion by their engagement with a MHC class II molecule. With positive proliferation these peptides could induce a protective response against sporotrichosis. CFSE-labeled cells were stimulated with the synthesized peptides and the negative controls (DMSO and PBS). The peptides soluble in DMSO (ZR3 and ZR8) induce proliferation in *S*. *brasiliensis*-sensitized cells (Fig. 3C and D). DMSO, the negative control, was unable to promote proliferation (Fig. 3B). The vaccine potential of GP70 peptides has been observed using a different proteomic approach, against *S*. *globosa*^27^. Of the peptides soluble in PBS, the ZR4 peptide was the only one that induced high cell proliferation (Fig. 3G). ZR6 induces low proliferation (Fig. 3I). The other synthesized peptides, ZR1, ZR5 and ZR7, could not induce cell proliferation (Fig. 3F,H and J). The ZR4 peptide came from a hypothetical protein of a fungus proteome.

In fungal infections, a mixed TH1/TH17 immune response confers resistance through the secretion of cytokines such as IFN-γ, TNF-α and IL-17A, which activate neutrophils and macrophages for fungal killing and clearance^28^. In the proliferation assay, the ZR3 peptide induces higher levels of IL-17A and IFN-γ production (Fig. 3L and M). Although we observed positive proliferation by the ZR4 and ZR8 peptide, we did not observe increase in cytokines levels (Fig. 3L and M). The ZR1, ZR5, ZR6 and ZR7 have similar cytokines levels as PBS control group (Fig. 3L and M). This immune response profile is protective against sporotrichosis^27,29,30^. Taken together with the cell expansion and cytokine production, we demonstrated a strong T cell activation by the ZR3, ZR4 and ZR8 peptides and higher levels of IFN-γ and IL-17A by the ZR3 peptide.

### Protection by antigenic peptides against subcutaneous sporotrichosis in BALB/c mice

**Figure 4:**
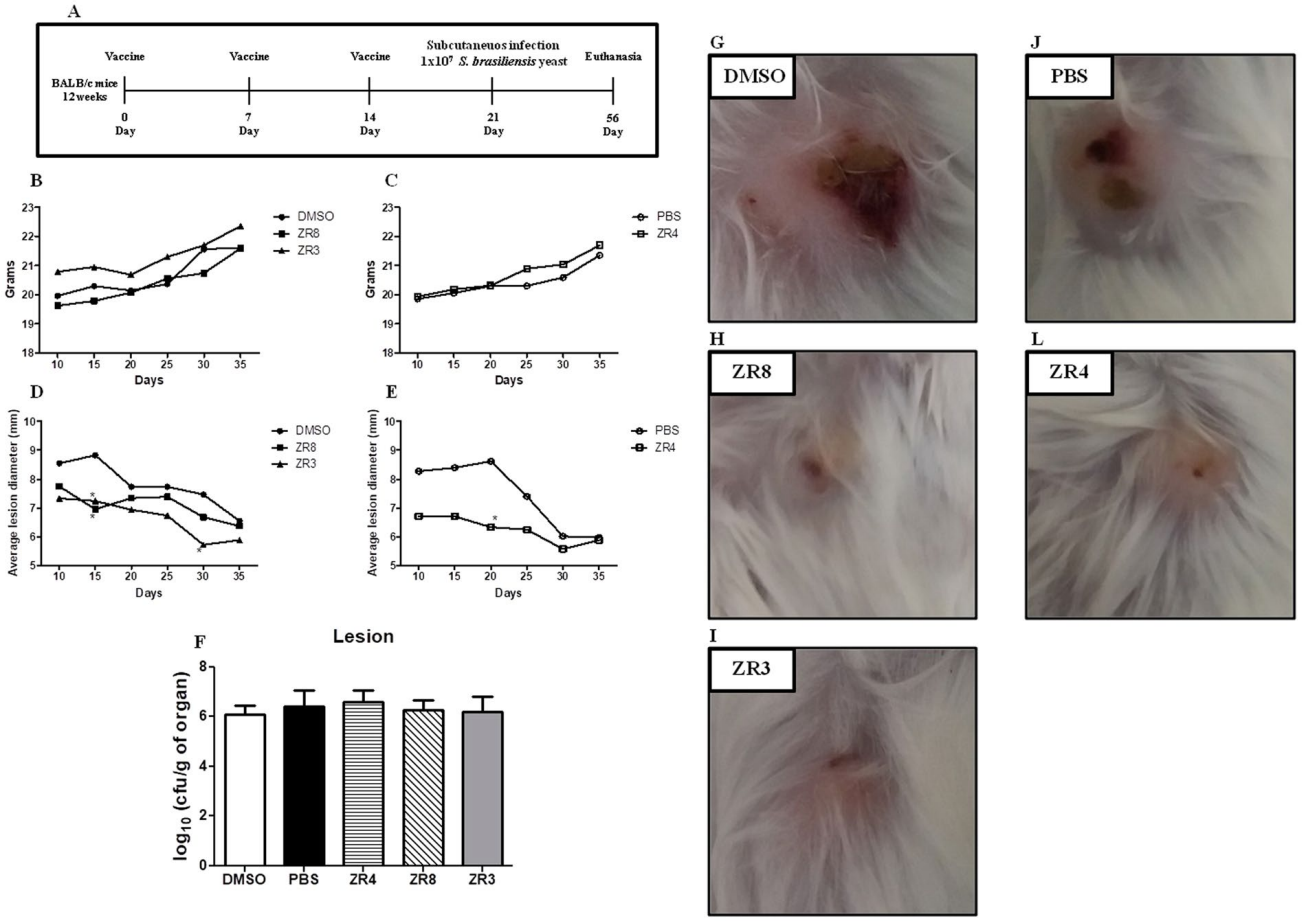
Subcutaneous sporotrichosis by *S*. *brasiliensis*. (**A**) Schematic representation of subcutaneous sporotrichosis model with the peptides vaccine. BALB/c female mice in the 7^th^, 14^th^ and 21^th^ days were inoculated 20 µg of peptide mixed with Freund’s adjuvant incomplete, in the ratio 1 to 1, in the leg. Mice were inoculated in the subcutaneous tissue 1 × 10^7^ of *S*. *brasiliensis* yeast cells. From the 10^th^ to the 35^th^ days post-infection, the average lesion diameter of the (**B**) DMSO group and of the (**C**) PBS group was measured. The statistical analysis was performed using Two-way ANOVA followed by Bonferroni post-tests. (**D**) After 35 days post infection, the lesion fungal burden was evaluated. (**E–I**) Pictures of lesion from the 15th day post infection.

A vaccine model was developed to test whether the synthesized peptides could induce a protective immunity against subcutaneous sporotrichosis (Fig. 4A). The progression of the disease was followed up to 35 days post-infection. The progression of subcutaneous sporotrichosis was determined by weight, the evolution of the primary skin lesion and the development of secondary lesions. No differences were observed in weight between the groups (Data not shown). All mice show a natural reduction in the lesion diameter on the 10^th^ to 35^th^ day post-infection (Fig. 4B and C). The ZR3, ZR4 and ZR8 peptides induce a reduction in the lesion diameter at the beginning of infection. The lesions were significantly smaller on the 15^th^ day post infection in ZR8-immunized mice and on the 15^th^ and 30^th^ day post infection in ZR3-immunized mice. ZR4 induces a significantly smaller lesion diameter on the 20^th^ day post infection. This reduction in the lesion diameter in subcutaneous sporotrichosis is shown from the 15^th^ day post infection (Fig. 4E to Fig. I).

The fungal burden was evaluated in lesions, liver, lymph nodes, spleen and kidney. No differences were observed in lesion CFU (Fig. 4D). We did not recover *S*. *brasiliensis* in other organs in immunized and control mice (data not shown). Although we did not observe disseminated sporotrichosis, all infected mice developed splenomegaly and lymphadenopathy, and the control group only developed arthritis. Similar secondary lesions were observed in different subcutaneous sporotrichosis models^14,31^.

### ZR8 and ZR3 peptides induced a protective immune response against sporotrichosis

**Figure 5:**
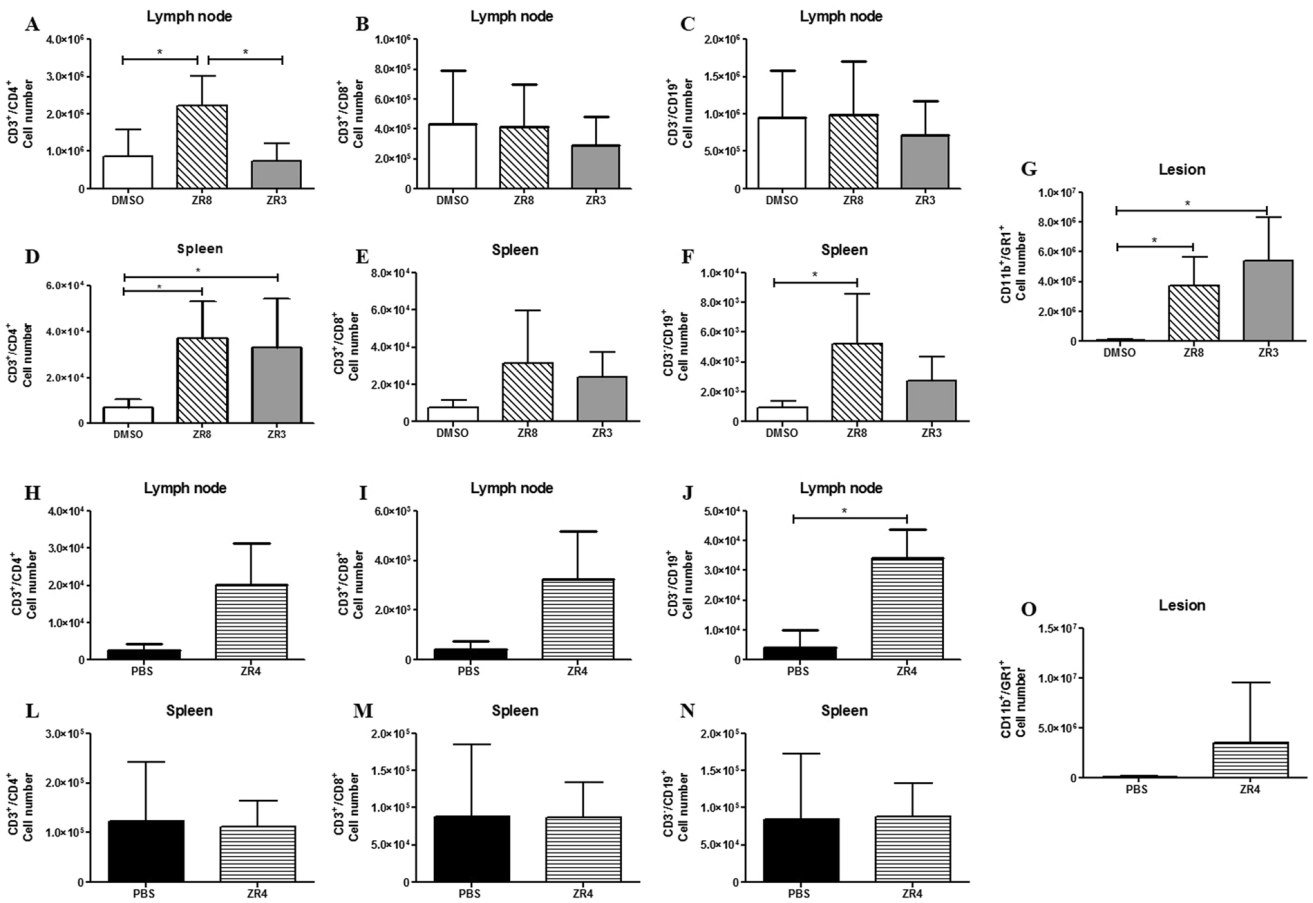
The protective immune response against sporotrichosis. To determine if the immunization with peptides induce a protective immune response were evaluated the cell profile by the cell number of CD3^+^/CD4^+^, CD3^+^/CD8^+^ and CD3^−^/CD19^+^ respectively in the DMSO group lymph node (**A–C**) and in the spleen (**D–F**). The cell profile of CD3^+^/CD4^+^, CD3^+^/CD8^+^ and CD3^−^/CD19^+^ respectively in the PBS group lymph node (**H–J**) and in the spleen (**L–N**). In the lesion was evaluated the cell profile GR1^+^/CD11b^+^ in the (**G**) DMSO group and in the (**O**) PBS group. Statistical analysis was performed using One-way ANOVA followed by Tukey’s test in DMSO group and t-test in PBS group.

We investigated whether the immunogenic peptides could induce a protective immune profile in subcutaneous sporotrichosis after 35 days of infection. The flow cytometry analysis revealed an increase in the cell numbers of CD3^+^/CD4^+^ in ZR8-immunized mice in the lymph nodes and spleen (Fig. 5A and D). The ZR3-immunized mice increased the number of CD3^+^/CD4^+^ cells in the spleen (Fig. 5D). The number of CD3^−^/CD19^+^ cells increased in the spleens of ZR8-immunized mice and in the lymph nodes of ZR4-immunized mice (Fig. 5F and J). The CD3^−^/CD19^+^ profile is associated with B cells. We did not observe a difference in the CD3^+^/CD8^+^ cell numbers in any groups (Fig. 5B,E,I and M). In the lesions in the ZR8- and ZR3-immunized mice, we observed an increase in CD11b^+^/GR1^+^ cell numbers (Fig. 5G). Neutrophils have a central role in fungal elimination through fungistatic and fungicidal responses.

**Figure 6:**
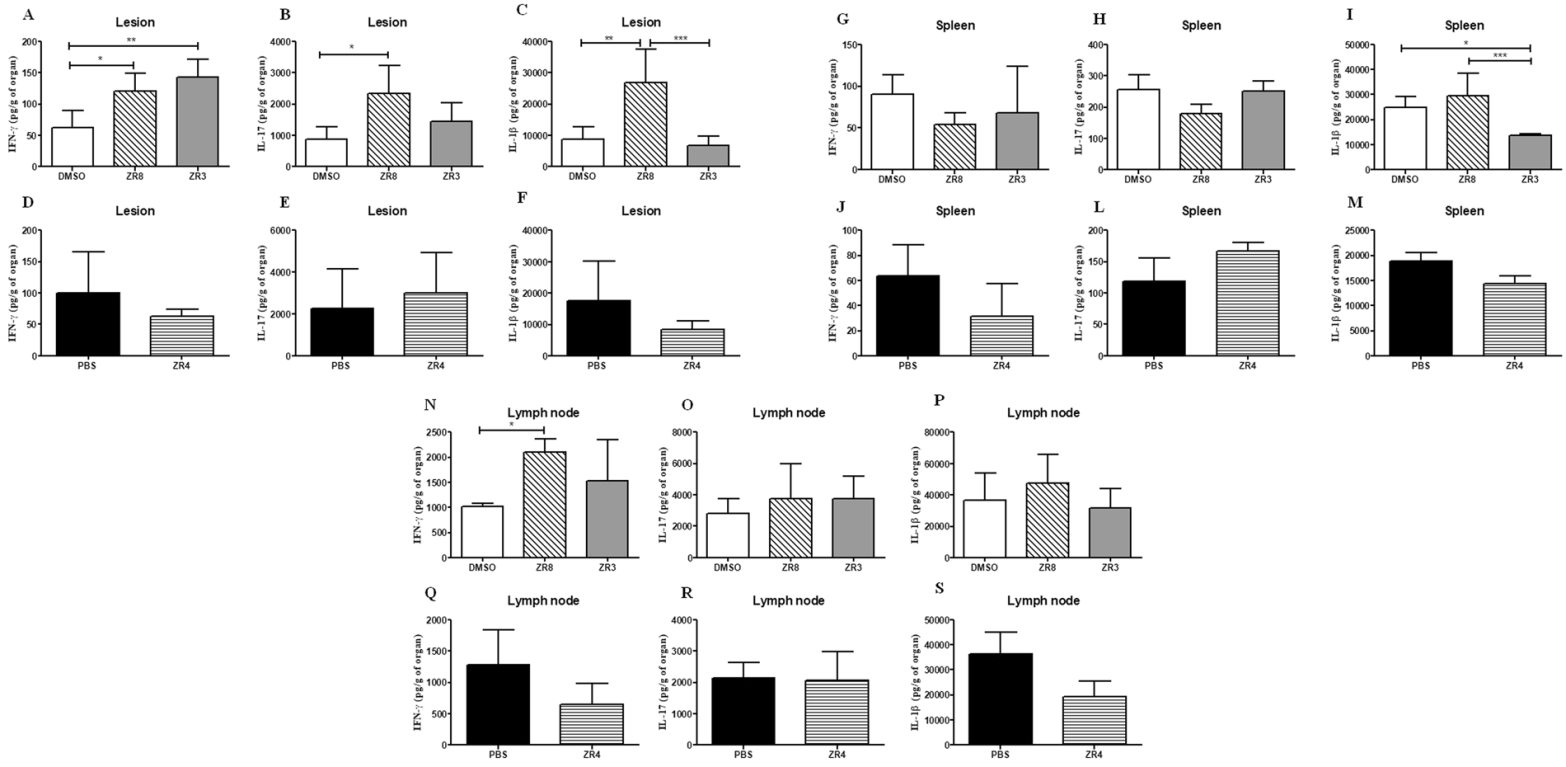
The cytokines levels induced by ZR3, ZR4 and ZR8 peptides in the subcutaneous sporotrichosis. To determine the cytokines levels of IFN-γ, IL-17A and IL-1β in the subcutaneous sporotrichosis were used ELISA assay kits (R&D Systems). These cytokines were determined in the (**A–F**) lesion, (**G–M**) spleen and in (**N–S**) lymph node. Statistical analysis was performed using One-way ANOVA followed by Tukey’s test in DMSO group and t-test in PBS group.

To determine the immune profile induced by peptides, we evaluated the IFN-γ, IL-17A and IL-1β levels in the lesions, lymph nodes and spleens. ZR8 peptide increases all these cytokine levels in the lesions (Fig. 6A,B and C). ZR8-immunized mice have higher IL-1β levels in the spleen and increased IFN-γ in the lymph nodes (Fig. 6I and N). ZR3 peptide induces high IFN-β levels in the lesions (Fig. 6A). ZR4-immunized mice have similar cytokine levels as the control group (Fig. 6N to S). A TH1/TH17 immune response is protective in sporotrichosis^27,32,33^. It was previously shown that the absence of IL-1β harms a protective adaptive immune response in sporotrichosis caused by *S*. *schenckii*^34^. Here, we observe that ZR8 peptide induces a protective cellular immune response with higher levels of IFN-γ, IL-17A and IL-1β.

## Discussion

Currently, fungal infections have become an emerging group in infectious diseases. Some conditions, such as AIDS, the increased use of chemotherapy in cancer treatment, and other factors that decrease the host immune response along with invasive hospital procedures, such as catheter use, have significantly increased cases of disseminated fungal infections^20,35,36^. Antifungal drugs are mycosis treatments more often used in immunocompetent and immunosuppressed patients. However, drug resistance in fungal infections has been reported for almost all antifungal drugs^37^. A vaccine that induces a protective immune response against fungi, with or without an antifungal drug, could promote a resistance in the host and could be a better approach against fungal infection. However, the development of vaccines against fungal infections is still a challenge. The relative genetic complexity of eukaryotic fungal cells limits knowledge about immune protection of the host against fungi^20^. In this study, we propose an immunoproteomic approach to selected immunogenic peptides from *S*. *brasiliensis* to develop a vaccine against subcutaneous sporotrichosis.

Sporotrichosis is endemic in Rio de Janeiro^4^. The recently described species *S*. *brasiliensis* is the most diagnosed species in these cases and the most virulent of the *Sporothrix* complex^8^. Using whole yeast cells and through engaging the MHC class II prediction programs, we synthesized 7 potential vaccine peptides. Three peptides were capable of inducing proliferation *in vitro* (ZR3, ZR4 and ZR8). ZR3 peptide is a sequence of the importin protein sequenced on the 2D gel (Table 1). A protein with metabolic functions, such as the transport of protein molecules to the nucleus, is present in several fungi. Proteins with metabolic activities that are widely distributed in other fungi may be the target of a fungal vaccine^20,38,39^. A vaccine with a metabolic heat shock fungus protein conferred protection against experimental histoplasmosis^40^. ZR3 peptide was the only one to induce higher levels of IFN-γ and IL-17A *in vitro*. Vaccine candidates against paracoccidioidomycosis, aspergillosis and blastomycosis induced cell expansion *in vitro*^20,41–43^. In sporotrichosis, GP70 is the most antigenic protein from the *Sporothrix* complex. A potential vaccine candidate against disseminated sporotrichosis caused by *S*. *globosa* is a peptide from GP70^27^. We used a GP70 peptide (LKFLALASVISATSA) called ZR8, which is the most promising peptide for a vaccine candidate for subcutaneous sporotrichosis.

In general, the CD4^+^ T cells by secreting the cytokines IFN-γ, TNF-α, and IL-17A determine host resistance to severe fungal infections, such as paracoccidioidomycosis, coccidioidomycosis, aspergillosis and candidiasis^20,32^. An effective vaccine is associated with CD4^+^ T cells^20,36^. A TH1 response activates macrophages by IFN-β that increases fungal killing and clearance and is considered the cytokine most important for sporotrichosis protection^33^. TH17 cells are involved in the activation and repair of epithelial barriers by IL-17A production, and are crucial for the antifungal defense and control of the NK cells^28,44^. Our results indicate that the ZR8 and ZR3 peptides induce a protective immune response against subcutaneous sporotrichosis caused by *S*. *brasiliensis*. The ZR3, ZR4 and ZR3 peptides as a vaccine decreased the lesion diameter, this smaller lesion diameter in immunized mice demonstrated a good prognosis in subcutaneous sporotrichosis. In different subcutaneous sporotrichosis models, *S*. *brasiliensis* is always the most virulent species inducing the worst injuries^14,31^. The ZR3 and ZR8 peptide increased CD4^+^ T cells in the spleen and lymph nodes (only ZR8) with higher numbers of neutrophils in the lesions. In addition, ZR8 peptide increases the levels of IFN-β and IL-17A in the lesions and IFN-β in the lymph nodes, suggesting a TH1/TH17 immune response profile.

The humoral immune response is very important in fungal infections. Many factors, such as opsonization and Fc receptors from phagocyte cells, promote a protective mechanism of the antibody immune response^45^. ZR8-immunized mice increase the CD3^−^/CD19^+^ cells in the spleen. Our group has already demonstrated the protective role of the antibody and humoral immune response against sporotrichosis and that the GP70 is associated with humoral response activation^16,26^. This response is protective in sporotrichosis by the presence of antibodies against GP70 and higher levels of IFN-γ^16,18,26^.

Through a different immune approach, Chen and collaborators revealed a potential novel vaccine candidate against *S*. *globosa* using a recombinant phage with a peptide from GP70. This recombinant phage increases TH1 cells and induces a strong humoral response that decreases the fungal burden in systemic sporotrichosis caused by *S*. *globosa*^27^. We did not observe a difference in the fungal burden of immunized and control mice, although the protective cellular immune response was promoted. Some factors, such as the time of infection, adjuvants and the *Sporothrix* form in infection, could modify sporotrichosis progression. In fungal vaccine approaches, a tetramer against blastomycosis was used as an adjuvant and a phage or PGA against sporotrichosis to observe CFU reduction^20,27,46^. In a sporotrichosis subcutaneous model with a yeast or conidial form, the infection time changes the fungal burden^14,31^. Thus, we believe that changes in some of these factors can be improved to observe a reduction in fungal burden in immunized mice since the peptides reduce lesion diameters.

In our study, the strain utilized was *S*. *brasiliensis* 5110 (ATCC MYA 4823) that was isolated from an infected cat in a sporotrichosis case at Rio de Janeiro. In these zoonotic sporotrichosis cases the disease tends to develop disseminated and severe forms^47–49^. In zoonotic transmission, the infected cats inoculate high loads of *S*. *brasiliensis* yeast by scratching or biting deeply into the tissue^50^. This differs from the sapronotic cases that involve the conidia of *S*. *schenckii* or *S*. *globosa*^11^. The inoculation of the yeast form is an important factor in the severity and gravity of the *S*. *brasiliensis* cases^51^. The yeast form of *S*. *brasiliensis* is adapted to the subcutaneous tissue, and it is very difficult for the immune system alone to effect the fungal clearance. An association with antifungal drugs or different adjuvants could decrease the fungal burden in our vaccine approach.

In conclusion, from 34 antigenic proteins identified from *S*. *brasiliensis*, 3 peptides were selected that induce proliferation in sensitized cells *in vitro*. We demonstrated that the ZR8 and ZR3 peptides induce a protective immune response against sporotrichosis. Although we did not observe any difference in the lesion fungal burden, the ZR8 and ZR3 immunized mice exhibited a protective immune response against sporotrichosis mediated by CD4^+^ T cells. With higher levels of IFN-β, IL-17A and IL-1β and increased numbers of CD4^+^ T cells in the lymph nodes and spleen, ZR8 peptide is the best vaccine candidate against subcutaneous sporotrichosis. With a longer time of infection in this protective immune response environment or with the addition of antifungal drugs, we would probably observe a decreased fungal burden in immunized mice. These data show improved advances in the immune approaches to an anti-fungal vaccine development and identified the ZR8 and ZR3 peptides as vaccine targets for the treatment of subcutaneous sporotrichosis.

## Material and Methods

### Mice

Female BALB/c mice at 10 weeks of age were obtained from the Animal House Production and Experimentation Facility of the Faculty of Pharmaceutical Sciences and Institute of Chemistry of the University of São Paulo. The mice were maintained in a SPF environment (specific pathogen free) and housed in temperature controlled rooms at 23–25 °C with free access to food and water throughout the experiments. Care and Research at the Faculty of Pharmaceutical Sciences (CEUA/FCF Protocol 513/16). This study was carried out in accordance with the recommendations of Guide for the Care and Use of Laboratory Animals of the National Institutes of Health. The protocol was approved by the Brazilian Conselho Nacional de Controle da Experimentação Animal (CONCEA).

### Microorganisms and Culture Conditions

The strain used was *Sporothrix brasiliensis* 5110 (ATCC MYA 4823). The strain was maintained by regular passages in animals and was grown on BHI agar (KASVI) at 37 °C.

### Protein extraction

The *S*. *brasiliensis* proteins were extracted according to Fonseca and collaborators with modifications^52^. *S*. *brasiliensis* proteins in the yeast phase were washed three times in ultrapure water by centrifugation at 5,000 × g for 5 min (4 °C). The pellet was macerated with liquid nitrogen and a pestle into a slim powder. The proteins were suspended in 5 mL of rehydration solution (7 M Urea, 2 M Thiourea, 4% Chaps, 0.4% Triton, 20 mM DTT and 0.5% Pharmalyte) and were vortexed for 10 min. The yeast debris was removed by centrifugation (11,000 × g, 4 °C, 10 min). Protein concentrations were determined by Bradford assay and the samples were kept at −80 °C until use.

### Two-dimensional gel electrophoresis

A final volume of 125 μl with 250 μg of *S*. *brasiliensis* proteins was added onto Immobiline DryStrip gel, linear pH 3–10 gradient, 7 cm strips (GE Healthcare) by overnight rehydration. The Immobiline DryStrip was focused out in an Ettan IPGphor 3 under the following sequential steps: Step 200 Vhr; Grad 300 Vhr; Grad 4000 Vhr; and Step 1250 Vhr. The strips were equilibrated with 1% DTT followed by 2.5% iodoacetamide at 15 min each, in SDS equilibration buffer (urea 6 M, Tris–HCl, pH 8.8, 75 mM, glycerol 29.3% [v/v], SDS 2% [w/v], and trace bromophenol blue). The second dimension was performed on a 12.0% polyacrylamide gel with a Tris–glycine buffer system. Equilibrated strips were placed on polyacrylamide gels and sealed with 0.5% agarose and separated. Gels were stained with the commercial Coomassie staining PhastGel Blue R-350 (GE Healthcare) and the Silver Staining Kit (GE Healthcare). Stained gels were digitalized using an ImageScanner III and the LabScanTM software (v6.0, GE Healthcare). Images were processed using the ImageMaster 2D Platinum software for protein spots enumeration.

### Serum for Western blot

Two groups of five Balb/c mice each were inoculated through the intraperitoneal (i.p.) route with 5 × 10^6^ yeast cells of *S*. *brasiliensis* 5110 suspended in 0.1 mL of sterile PBS or with sterile PBS. After 25 days of infection, the five mice in each group were euthanized in a CO_2_ chamber to draw mouse blood by intracardiac puncture. Serum samples were obtained by centrifugation (10,000 × g, 4 °C, 10 min) and kept at −80 °C until use.

### 2D immunoblot of *Sporothrix brasiliensis* proteins

The *S*. *brasiliensis* proteins on 2D gel were transferred to Hybond ECL nitrocellulose membranes (GE Healthcare) by a TE 70 PWR Semi-dry transfer unit (GE Healthcare) at 60 mA for 1 h with transfer buffer (25 mM Tris base, 192 mM glycine, 20% methanol; pH 8.3). The success of electrotransference was evaluated by Ponceau S (Ponceau S 0.15% and acetic acid 1%) staining. The membranes were washed, and the free binding sites were then blocked for 2 hours in PBS blocking buffer (1% bovine serum albumin, supplemented with 0.05% Tween and 20.5% skim milk) at room temperature. The membranes were probed with 20 µg of mAbP6E7 or pooled serum from mice, infected or not with *S*. *brasiliensis*, by a dilution of 1:200 at 8 °C overnight. Subsequently, the membranes were washed three times with PBS (pH 7.5) containing 0.05% Tween-20 (PBS-T) for 10 min and incubated with peroxidase (HRP)-conjugated goat anti-mouse IgG (1:1000 dilution) for 2 hours at room temperature. Finally, the membranes were washed and developed using the ECL Plus Kit (GE Healthcare).

### Identification of seroreactive proteins by MALDI–ToF MS/MS

MS analyses were performed at CEFAP-USP (Core Facility for Scientific Research, University of São Paulo). Briefly, the spots of interest were manually excised (16 spots) from the four stained Coomassie gels. They were destained in 25 mM NH_4_HCO_3_ in 50% acetonitrile solution followed by treatment with 100% acetonitrile solution. Proteins were digested with 12.5 ng/L sequencing grade trypsin (Roche Molecular Biochemicals) in 25 mM NH_4_HCO_3_ overnight at 37 °C. The digested material was collected and desalted in Milipore Zip-Tip C18 pipette tips. After trypsin digestion, the peptide suspension derived for each spot was spotted onto a MALDI target plate, mixed with matrix α-cyano-4-hydroxy-trans-cinnamic acid (Sigma), and allowed to dry at room temperature. The samples were analyzed on an Autoflex MALDI-TOF/TOF mass spectrometer (Bruker) with the Bruker Daltonics flexAnalysis program. The four most intense peaks were selected and analyzed by Pattern Lab Proteomics for protein identification^53^. The database from *S*. *brasiliensis* 5110 was used^25^. The contaminant database and analysis procedures were used in accordance with the system. The search parameters included the variable modification oxidation (M) and the fixed modification carbamidomethyl (C). Up to one missed cleavage site was allowed; the mass tolerance of the peptide was 0.05 Da, and the MS/MS tolerance was 0.2 Da. A false discovery rate (FDR) of 1% was applied.

### Epitope identification

After the protein identifications were analyzed, predictions were made of the affinities of the peptides to MHC class II. All peptide sequences from identified proteins were analyzed by the Immune Epitope Database (IEDB) Analysis Resource and PREDBALB/c^54-56^. In the IEDB Analysis Resource, the program tools consider the lower number to indicate the greater affinity, where values <50 nM are considered to be high affinity, <500 nM are considered of intermediate affinity and <5000 nM are considered of low affinity. The sequences with high and intermediate affinity scores (<50 nM and <500 nM, respectively) were selected. In PREDBALB/c, the higher number means higher affinity with MHC class II; the values range from 0 to 10, and for the analysis the peptides with values equal or higher than 9.5 were chosen. We chose the peptides with the best scores in both programs. Since GP70 is an important antigen from the *Sporothrix* complex, we also chose the sequence from this protein with the best scores in both programs. The peptides were synthesized by Life Technologies.

### Analysis of mouse T cell responses

To evaluate T cell proliferation, five Balb/c mice were intraperitoneally infected with 5 × 10^6^ yeast cells of *S*. *brasiliensis* 5110 suspended in 0.1 mL of sterile PBS. The spleens were removed from the mice 15 days after infection. The total cells from the spleens were stained with 5(6)-carboxyfluorescein diacetate N-succinimidyl ester (CFSE) in accordance with the KIT protocol. The spleen cells (3 × 10^5^) were cultivated in supplemented R10 medium (1% non-essential amino acids – MEM NEAA (Gibco) +1 mM sodium pyruvate (Gibco)). The cells were stimulated with 5 micrograms of the synthesized peptides that were soluble in PBS (ZR1, ZR4, ZR5, ZR6 and ZR7) as a negative control PBS and with peptides that were soluble in DMSO (ZR3 and ZR8) as a negative control DMSO. Phytohemagglutinin (PHA) was used as a positive control. The plate was incubated at 37 °C with 5% CO_2_ for 5 days. The reading was performed on the flow cytometer (FACSCantoII, BD) and analysis in FlowJo software.

### Immunizations

Mice were immunized intramuscularly with 20 µg of each peptide mixed with Freund’s incomplete adjuvant (Sigma) in the ratio of 1 to 1. Immunization was repeated 3 times every 7 days. PBS and DMSO were used as negative controls.

### *In vivo* infection

Groups of 5 mice each were infected by the subcutaneous route with 1 × 10^7^ *S*. *brasiliensis* 5110 yeast cells diluted in PBS 7 days after the last immunization. The mice were euthanized 35 days after infection. The lesions, livers, spleens, kidneys and lymph nodes from each animal were homogenized in PBS and the fungal burden was determined by CFU assay in BHI agar (Kasvi) and incubated at 25 °C for 7 days. Recovered colonies were counted, multiplied by the dilution factor and expressed as log means (CFU/gram of organ). Organ homogenates were then centrifuged at 3000 rpm for 10 minutes and the supernatants were collected for cytokine measurements.

### Cell profiles

We evaluated the cell profile in the lesion, spleen and lymph nodes from infected mice. To determine the CD3^+^/CD4^+^, CD3^+^/CD8^+^ and CD3^−^/CD19^+^ in the spleen and lymph nodes cells we used labeled mAbs against mouse PE CD4 (GK 1.5), FITC CD3 (145–2C11), PE-Cy7 CD8 (53–6.7) and APC-Cy5 CD19 (1D3). To determine the GR1^+^/CD11b^+^ in the lesion cells, we used labeled Mabs against mouse APC GR1 (RB6–8C5) and FITC CD11b (m1/70). All antibodies were obtained from BD Biosciences, San Jose, CA. The flow cytometry data were analyzed using FlowJo. Fluorescenceminus-one (FMO) tubes were used as additional controls.

### Cytokine measurements

Cytokine levels were measured with an ELISA assay (R&D Systems kits) according to the manufacturer’s protocol. The cytokines assayed were IL-1β, IFN-γ and IL-17A.

### Statistical analysis

Prism 5 software (GraphPad Software Inc, LA Jolla, CA) was used for all tests and differences were considered significant when p ≤ 0.05. The results are expressed as mean standard error (SD). Statistical analysis was performed using analysis of variance (ANOVA) followed by the parametric Tukey Kramer test (INSTAT software: GraphPad, San Diego, CA, USA) and One-way ANOVA followed by Tukey’s test were used to calculate statistical significance (p-values).

## Electronic supplementary material

Supplementary Information
