# Supplementary material for: An immunoproteomic approach revealing peptides from Sporothrix brasiliensis that induce a cellular immune response in subcutaneous sporotrichosis
Source: Sci Rep. 2018 Mar 8;8:4192. doi: 10.1038/s41598-018-22709-8 (PMC5843658; doi:10.1038/s41598-018-22709-8)
Supplement: Supplementary file 1 — Supplementary Information [file 41598_2018_22709_MOESM1_ESM.pdf]

An immunoproteomic approach revealing peptides from *Sporothrix brasiliensis* that induce a cellular immune response in subcutaneous sporotrichosis

José Roberto Fogaça de Almeida,[1] Grasielle Pereira Jannuzzi,[1] Gilberto Hideo Kaihami,[2] Leandro Carvalho Dantas Breda,[3] Karen Spadari Ferreira,[4] Sandro Rogério de Almeida[1]*

[1] Faculty of Pharmaceutical Sciences, Department of Clinical and Toxicological Analysis, University of Sao Paulo, Sao Paulo, Brazil
[2] Institute of Chemistry, Department of Biochemistry, University of Sao Paulo, Sao Paulo, Brazil
[3] Department of Immunology, Institute of Biomedical Sciences, University of São Paulo, São Paulo, Brazil.
[4] Department of Biological Sciences, Institute of Environmental, Chemical and Pharmaceutical Sciences, Federal University of São Paulo, Diadema, Brazil.

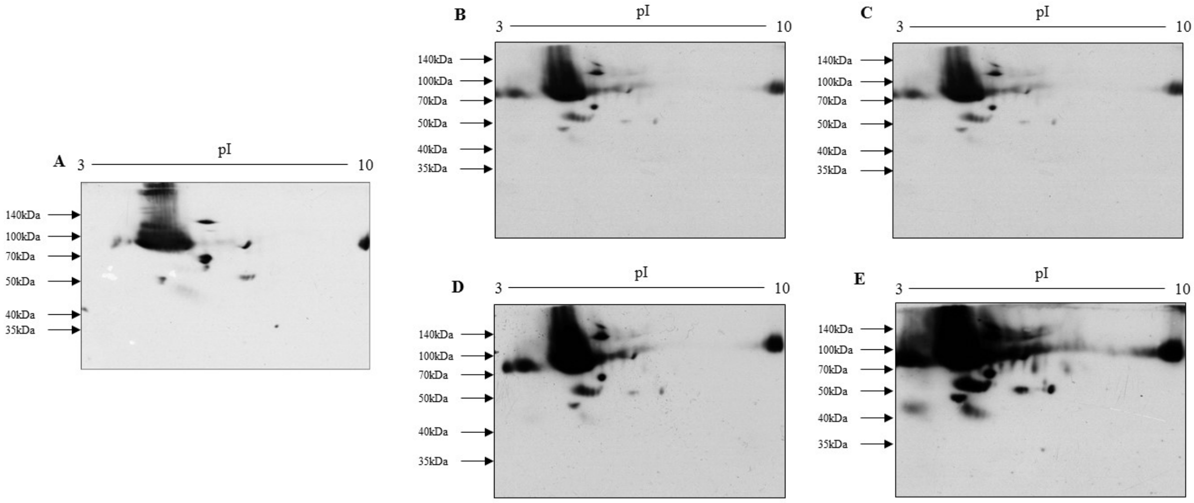

Supplementary figure 1. The multiple exposure of *S. brasiliensis* proteome western blot. (**A**) Proteome of *S. brasiliensis* 5110 (ATCC MYA 4823). The fungus proteins were fractionated using 7 cm pH 3–10 (left to right) strips in the first dimension and 12% SDS-PAGE gels in the second dimension, the spots recognized by western blot with sera from mice infected by *S. brasiliensis*. (**B-E**) Different overexposure of the western blot with sera from mice infected by *S. brasiliensis*.

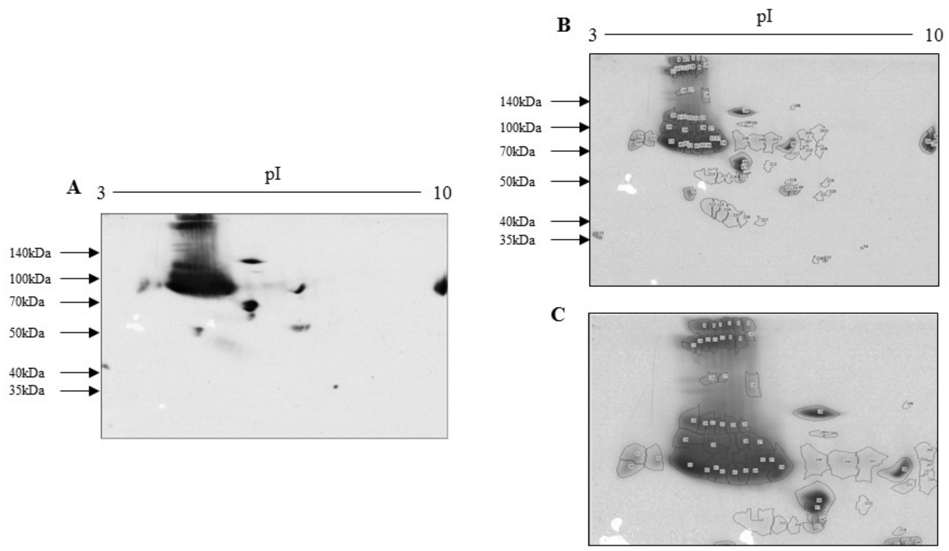

Supplementary figure 2. ***S. brasiliensis*** **proteome western blot analyzed by ImageMaster 2D Platinum 7.0.** (**A**) Proteome of *S. brasiliensis* 5110 (ATCC MYA 4823). The fungus proteins were fractionated using 7 cm pH 3–10 (left to right) strips in the first dimension and 12% SDS-PAGE gels in the second dimension, the spots recognized by western blot with sera from mice infected by *S. brasiliensis*. (**B**) The total spots of the gel surrounded borders by a black line in the ImageMaster 2D Platinum 7.0 (GE Healthcare). (**C**) Until 50kDa spots of the gel, where is the most spots number, surrounded borders by a black line in the ImageMaster 2D Platinum 7.0 (GE Healthcare).
